# The Role of Transoral Robotic Surgery in the Management of Oropharyngeal Cancer: A Review of the Literature

**DOI:** 10.5402/2012/945162

**Published:** 2012-04-23

**Authors:** Samuel A. Dowthwaite, Jason H. Franklin, David A. Palma, Kevin Fung, John Yoo, Anthony C. Nichols

**Affiliations:** ^1^Department of Otolaryngology-Head and Neck Surgery, The University of Western ON, London, ON, Canada N6A 3K7; ^2^Department of Radiation Oncology, The University of Western Ontario, London, Ontario, Canada N6A 3K7; ^3^Victoria Hospital, London Health Science Centre, Department of Otolaryngology-Head and Neck Surgery, Room B3-438A, 800 Commissioners Road East London, ON, Canada N6A 5W9

## Abstract

*Background*. Transoral robotic surgery (TORS) is an emerging treatment option for the treatment of head and neck malignancies, particularly for oropharyngeal squamous cell carcinoma (OPSCC). Preliminary studies have demonstrated excellent oncologic and functional outcomes that have led to a resurgence of interest in the primary surgical management of OPSCC. The aim of the present study was to review the evidence base supporting the use of TORS in OPSCC. *Methods*. Studies evaluating the application of TORS in the treatment of head and neck squamous cell carcinoma (HNSCC), and more specifically OPSCC, were identified for review. Further searches were made of reference lists for complete evaluation of minimally invasive surgery (MIS) in treating OPSCC. *Results*. Seventeen results relating to the application of TORS in treatment of OPSCC were identified. Further results relating to the role of transoral laser microsurgery (TLM) in OPSCC were included for review. Feasibility, oncologic, and functional data is summarized and discussed. *Discussion*. Management strategies for patients with OPSCC continue to evolve. Minimally invasive surgical techniques including TORS and TLM offer impressive functional and oncologic outcomes particularly for patients with early T-classification and low-volume regional metastatic disease. Potential exists for treatment deintensification, particularly in patients who are HPV positive.

## 1. Introduction

Approximately 500,000 new cases of head and neck squamous cell carcinoma (HNSCC) are diagnosed each year making HNSCC the 6th most common cancer worldwide [1]. The rate of HNSCC has been increasing recently secondary to an epidemic of human papillomavirus- (HPV-) related oropharyngeal squamous cell cancer (OPSCC).

These trends, which have seen rates of OPSCC as much as double over the last three decades in some countries, have been associated with a shift in demographics to a younger population that is typically high functioning with lower rates of comorbid illness [2]. Combined with lower rates of smoking and alcohol abuse and an intrinsic improved response rate of HPV-related OPSCC to curative treatment regimens, the overall and disease-free survival rates in this patient group are significantly higher than previously observed. As such, long-term quality of life considerations have been made even more relevant to the multidisciplinary team coordinating the care of these patients.

These new developments in the pathogenesis of HNSCC, and in particular OPSCC, are occurring in the context of large-scale paradigm shifts over the past two decades towards organ preservation treatment protocols. The Veterans Affairs laryngeal cancer study published in 1991 began the era of organ preservation and led to the application of primary radiation approaches to other subsites such as the oropharynx [3]. Although a randomized controlled trial of primary radiation versus surgery for oropharyngeal cancer has not been carried out, several retrospective analyses have demonstrated less major complications and improved functional outcomes with radiation compared to traditional primary surgical approaches. These results have contributed to the widespread adoption of primary nonsurgical treatment in patients with OPSCC.

Minimally invasive surgery (MIS) for HNSCC continues to be extensively reported within the head and neck literature, driven by the desire to offer a less morbid alternative to chemoradiation. These techniques have included transoral laser microsurgery (TLM) and more recently, transoral robotic surgery (TORS). TORS was first introduced into the literature by Weinstein et al. in 2005 with their case report of a supraglottic laryngectomy performed in a canine model [4]. The adaptation of this preexisting robotic technology to a new clinical application provided a watershed moment in the evolution of treatment options for the surgical management of head and neck aerodigestive tract pathology. 

Since this early report, the development of TORS in its various human applications has been steadily progressing with feasibility studies confirming the safety and usability of this technology in live human patients [5, 6]. Given the benefit of infield optics provided by the robot-mounted double video endoscope, line-of-sight issues associated with more traditional transoral techniques are overcome with the use of TORS. This factor, combined with the valuable 3-dimensional imagery, tremor filtration and the resultant precision movements translated from the console to the robotic instruments at the operative site, allow for accurate dissection of tissue planes in a way not previously possible.

Various reports of the use of TORS in benign disease processes have been published in the literature [7–10], however, the majority of reports have concentrated on the application of TORS in patients with mucosal malignancies, particularly of the larynx and oropharynx [11–14]. Emerging evidence suggests that effective primary surgical management of these malignancies may provide an opportunity for deintensification of adjuvant treatments with resultant improvements in patient's posttreatment quality of life, without compromising oncologic outcomes.

The aim of the current review is to provide an evaluation of the existing literature with regards to the oncologic and functional outcomes following treatment of OPSCC with minimally invasive surgery and in particular, TORS.

## 2. Methods

A literature search was conducted using PUBMED and MEDLINE with search parameters including (transoral[Title] AND ((“robotics”[MeSH Terms] OR “robotics”[All Fields] OR "robotic"[All Fields]) OR robot[All Fields] OR telerobot[All Fields] OR (“robotics”[MeSH Terms] OR “robotics”[All Fields] OR “telerobotics”[All Fields]) OR robot-assisted[Title])) OR TORS[Title].

The references of the identified studies were reviewed further for completeness. In addition, studies relating to the use of TLM were identified to facilitate a complete evaluation of the evidence base for the current role of MIS in the management of OPSCC.

Only those studies dealing specifically with the management of patients with HNSCC were included, with descriptions of TORS for benign disease, nonmucosal malignancies of the upper aerodigestive tract, novel applications of TORS and descriptions of cadaveric studies all being excluded. Specifically, data relating to the evaluation of quality of life, oncologic and functional outcomes in the identified studies was reviewed.

## 3. Results

Sixty-nine results were identified for review of which the references were further evaluated for potential inclusion in the analysis. Of particular note, studies related to the use of transoral laser microsurgery were included for complete review of minimally invasive surgery in the treatment of OPSCC.

Sixteen studies were excluded as they were not related to the application of TORS in patients with HNSCC. Forty studies addressed the role of TORS in treatment of head and neck tumors and in particular, 17 studies commented directly on the use of TORS in patients with OPSCC. Six studies were identified that investigated quality of life and functional outcomes following TORS for treatment of HNSCC/OPSCC with a further seven discussing feasibility aspects including setup and operative times (Table 1).

### 3.1. Feasibility

The initiation of new TORS programs, generally established in conjunction with robotic programs in other surgical specialties, has repeatedly demonstrated success in establishing safe and efficient robotic services for head and neck oncology units [15–19]. Operative setup times have been reported to take between 2 minutes and 140 minutes. Generally, average setup times after preliminary experience within the TORS team are under 30 minutes. Further demonstration of the increased efficiency following the introduction of TORS to a department is documented in the data from Moore et al. who significantly improved the setup time after their initial experience, suggesting that the learning curve for operative efficiency is complete after approximately 10 patients [14]. The average operative time across all 7 studies is just under 75 minutes.

For patients who require free flap reconstruction of the ablative defect, feasibility studies and initial case series detailing free tissue transfers via minimally invasive surgical approaches and robotic-assisted microanastomotic techniques have also demonstrated reliable perioperative outcomes [17, 20–25].

### 3.2. Oncologic Outcomes

In excess of 500 patients receiving TORS to manage OPSCC have been reported across 17 studies. A summary of the TORS literature relating to the oncologic outcomes in patients with OPSCC is presented in Table 2. Generally, these studies are retrospective in nature and report on highly selected patients. Many reflect treatment of advanced stage disease by virtue of N-positivity with associated early T-classification: several trials report only on the treatment of T1-2 lesions with various stages of cervical disease [12, 16, 26, 27]. These factors make generalizations and direct comparisons to the nonsurgical literature difficult.

Nonetheless, preliminary data relating to local control, disease-specific survival, and overall survival using upfront TORS is encouraging, with early overall survival rates at 1 year exceeding 90% with emerging 2-year survival data between 80% and 90% [13, 20, 28, 29]. Cohen et al. reported 1 and 2 year overall survival rates among a 50 patient cohort of 95.7% and 80.6%, respectively. Thirty-nine patients had T1/T2 lesions (78%), 8 had T3 lesions (16%), and 3 had T4 lesions (6%), with this low primary tumor volume further demonstrated by the fact that only a single free tissue transfer was required for reconstruction of the ablative defect [13]. Ongoing efforts at other institutions have replicated these early survival results, with Genden et al. demonstrating an overall survival of 90% amongst their cohort of 30 patients treated with primary TORS for HNSCC, whilst White et al. recorded a 86.5% 2 year overall survival for 71 patients undergoing TORS as part of treatment for HNSCC of all subsites (87% patients had OPSCC) [20, 29]. In both of these studies, a majority of patients again had advanced stage disease with early T-stage status and small volume nodal disease.

These oncologic outcomes with TORS largely mirror the experience reported in the TLM literature with patient populations again largely demonstrating early T-classification, deintensified adjuvant treatment recommendations based on pathological staging, and impressive oncologic control (Table 3). Steiner was one of the first to report on this in 2003 with a report on 48 patients treated for mostly advanced stage OPSCC involving the base of the tongue demonstrating a local control rate over 5 years of 85% [30]. Most recently, Haughey et al. have published multicenter data on 204 patients treated with TLM ± adjuvant therapy for advanced stage OPSCC, with 2 and 5 year overall survival rates of 89% and 78%, respectively, and a local control of 97% [31]. Adjuvant treatment was avoided in 26%, with the remaining 150 patients receiving adjuvant treatment of which 33 required chemoradiotherapy. Although adjuvant radiotherapy was associated with improved overall survival, the addition of chemotherapy did not significantly impact on survival.

This potential to deintensify treatment based on successful surgical control of the primary tumor burden has led to retrospective analysis of the experience in some centers with single modality treatment of OPSCC. Grant et al. reported in 2009 their experience with 69 patients with select mostly early T-stage OPSCC who did not receive adjuvant therapy (64% not indicated and 36% offered but declined) [32]. The 5-year overall survival rate was 86% with no patients requiring either tracheostomy of gastrostomy tube.

### 3.3. Functional Outcomes

It remains unclear whether a subset of patients with OPSCC can be treated with primary surgery in order to achieve superior functional results with deintensification of adjuvant treatment. Following on from Steiner's early work the use of TLM has repeatedly demonstrated impressive functional outcomes with low rates of gastrostomy dependency, decannulation and high quality of life scores on questionnaire assessment. These results are summarized in Tables 3 and 4.

Early TORS data supports these impressive functional outcomes with most series reporting 1-year gastrostomy-tube rates under 10% and long-term rates of 0% for patients treated with upfront surgery [14, 20, 28, 33] (Table 5). Higher rates of gastrostomy tube dependency have been reported in TORS case series including all HNSCC subsites with typically higher swallowing dysfunction observed in patients with laryngeal malignancies [1, 2].

### 3.4. The Role of HPV

A further confounder in the management of OPSCC is the role of HPV in disease pathogenesis and significance as a prognostic factor, apparently irrespective of treatment modality [13, 34–36]. The exact incidence of HPV infection and its impact on patients with OPSCC is still being elucidated in prospective trials after having initially been appreciated in retrospective studies. Ang et al. published their experience in the treatment of 323 patients with locally advanced OPSCC [37]. The HPV-positive rate was 63.8% (206/323); HPV-positivity was associated with a significant relative reduction in the risk of death of 58%, translating to an 82.4% survival rate at 3 years compared to 57.1% in those patients HPV-negative. This significant survival advantage was also demonstrated Nichols et al. in 2010 reporting on 68 patients with OPSCC, of whom 53 were found to be HPV-positive (78%) [38]. Patients who were HPV-positive were 5 times less likely to develop recurrence and 60% less likely to die of disease when compared to their HPV-negative counterparts. 

Weinstein et al. also reported on 50 patients treated for OPSCC with an HPV-positive rate of 74% (37/50). In this series of patients treated with upfront TORS, the disease-specific survival of patients with HPV-positive OPSCC at 1-year and 2-years was 97% and 90%, respectively. However, the HPV-negative cohort in this primary surgical series demonstrated equal oncologic control with disease-specific survival rates at both 1-year and 2-years of 100%.

## 4. Discussion

The management of patients with OPSCC continues to evolve with both advances in therapeutic regimens and evolution of our understanding of the underlying disease pathophysiology. Early surgery-based treatment paradigms have been largely abandoned with the general adoption of nonsurgical regimens supported by an evidence base demonstrating superior functional outcomes whilst retaining comparable survival results [39–41].

Paralleling the improvements in surgical technique, the natural evolution of radiotherapy has resulted in a decrease in treatment-related morbidity. Refinement of targeted 3D conformal radiotherapy methods such as intensity-modulated radiotherapy (IMRT) has allowed increased precision in delivery of radiotherapy to patients with HNSCC with associated decreases in adverse sequelae [42]. Tempering these benefits, however, has effects related to escalation of treatments in an effort to improve survival outcomes. Meta-analysis data have demonstrated improved survival with altered fractionated regimens and/or the addition of chemotherapy, at the expense of significant increases in treatment related toxicities, particularly acute mucositis and long-term swallowing dysfunction [43–46].

As the etiologic factors involved in the development of OPSCC continue to be elucidated one variable that has proven immensely significant is the role of human papillomavirus (HPV). This HPV epidemic has interestingly occurred during the same period as the widespread adoption of chemoradiotherapy as standard of care treatment for advanced stage OPSCC. This has managed to blur the lines somewhat with respect to whether these improved outcomes are secondary to treatment modifications or reflective of a naturally improved survival rate in patients with HPV-positive disease. Furthermore, the HPV-positive OPSCC patient has been defined as typically younger with lower rates of significant medical comorbidities [47]. Combined with improved rates of cure this young, high functioning population requires special consideration given that any treatment related side effects may need to be lived with for extended periods of time.

Oncologic outcomes in patients with HPV-positive OPSCC remain similar regardless of treatment approach. Therefore, an appreciation of functional outcomes represents a significant and fundamental consideration when formulating management recommendations. Transfacial and transmandibular surgical approaches to the oropharynx carry potential morbidity and have been demonstrated on retrospective analysis to be associated with poor functional outcomes when compared to radiotherapy [39–41]. Both TLM and TORS facilitate surgical access to the lower subsites of the upper aerodigestive tract without need for traditional methods requiring open surgical approaches. Repeatedly, these MIS techniques have both demonstrated sound oncologic and functional/quality of life outcomes [14, 28, 48–52]. Potential benefits also exist with respect to adjuvant treatment deintensification and avoidance altogether in select circumstances [32, 53–55].

Comparisons of outcomes after TORS versus chemoradiotherapy across studies are hampered by differences in baseline patient populations, selection, and treatment technique. Nonetheless, rates of gastrostomy tube dependency after chemoradiotherapy have typically been reported as between 9% to 39% in patients treated with chemoradiotherapy [56–59]. Critical analysis of the chemoradiotherapy literature reveals a patient population that typically differs from the recent MIS literature with inclusion of patients with unresectable (T4b) and/or bulky primary disease (greater than 4 cm), and typical exclusion of early primary disease such as in the series reported by the Radiation Therapy Oncology Group [37]. Therefore, direct comparisons across these reported functional outcomes are difficult.

The promising impact of TORS on the quality of life and survival outcomes of OPSCC patients remains an important clinical question that requires higher levels of supporting evidence. A recent surgical consensus statement (the “IDEAL” guidelines) suggests that randomized comparisons be carried out wherever possible, once adequate pretrial data are available [60]. Through centres in London and Ottawa, Ontario, the ORATOR Study (Oropharyngeal cancer Radiation versus TORS) is planned to open in early 2012. This study will randomize OPSCC patients to curative intent treatment with either upfront TORS ± adjuvant treatment versus nonsurgical treatment, with the primary endpoint being superior swallowing function at one year in the TORS arm as determined by the MD Anderson Dysphagia Inventory (MDADI).

Ultimately, ORATOR aims to assist in the identification of OPSCC patients who might be best served by upfront surgery. Adapting data from other head and neck subsites we understand that in a select group of “surgical responders” chemoradiotherapy represents a suboptimal treatment approach due to either lack of response in locoregional disease control or excessive treatment-related side effects and morbidity. Further, defining the “surgical responder” in patients with OPSCC is perhaps even more important than other subsites such as the larynx given the relative lack of success with surgical salvage. Combined with the work of others, ORATOR can hopefully work to identify the ideal OPSCC patients for upfront TORS, with the realization that chemoradiotherapy will still represent an excellent treatment option for the “nonsurgical responder.”

Ultimately, providing truly accurate risk assessment will require a clearer understanding of the underlying molecular genetics of HNSCC. Only then can patients be risk stratified with relative certainty utilizing both clinical and genetic evidence to guide treatment plans. Recent high-throughput genetic sequencing publications in science by Agrawal and Stransky provide exciting insights into a future that promises genetically guided management to optimize survival and minimize treatment-related morbidity [61, 62]. Further, current vaccination programs aimed at reducing HPV transmission within the wider community provide an exciting opportunity to impact significantly on the incidence of OPSCC.

## 5. Conclusion

The continued development of minimally invasive surgical techniques such as TORS offers a significant opportunity to impact positively on patient quality of life and posttreatment function whilst retaining satisfactory oncologic control. Initial feasibility and case series reports are encouraging but require further validation through well-designed randomized control trials prior to widespread shifts in accepted treatment paradigms.

## Figures and Tables

**Table 1:** Setup and operative times for TORS.

Study	Complete setup time (minutes)	Operative time (minutes)
O'Malley et al. [12] (3 patients)	Ave—44	Ave—105
	Range—38–52	Range—91–131
Weinstein et al. [11] (27 patients)	Ave—9	Ave—103
	Range—2–22	Range—26–233
Genden et al. [16] (18 patients)	Ave—55	Ave—84
	Range—20–140	Range—45–150
Park et al. [27] (5 patients)	Ave—19	Ave—44
	Range—15–25	Range—40–50
Moore et al. [14] (45 patients)	Ave 1st 10–69	Ave—72
	Range—54–59	Range—45–320
	Ave 2nd 35–22	Ave—71
	Range—14–28	Range—6–309
Lawson et al. [19] (24 patients)	Ave—24	Ave—67
	Range—10–60	Range—12–180
Aubry et al. [15] (17 patients)	Ave—21 Range—10–50	Ave—40
		Range—10–90

		Average operative time: 75

**Table 2:** Oncologic outcomes following TORS for OPSCC.

Study	Number of cases	Primary site	Pathological stage	HPV status	Followup period (mean)	Oncologic outcomes
Cohen et al. [13] (Apr '10)	50	Oropharynx	T1/2: 39	HPV-neg: 13	HPV-neg: 23.0 mths	1 yr: 95.7% (45/47)
			T3: 11			2 yr: 80.6% (25/31)
				HPV-pos: 37	HPV-pos: 24.8 mths	
			N0: 9			
			N1: 21			
			N2a: 0			
			N2b: 20			
			N2c: 0			
			N3: 0			

Genden et al. [20] (Aug '11)	30	27 oropharynx (90%)	T1/2: 30/30	N/A	20.4 mths	18 mth:
		1 larynx (3.3%)				locoregional control: 91%
		1 oral cavity (3.3%)	N0: 6			distant control: 93%
		1 hypopharynx (3.3%)	N1: 10			DFS: 78%
			N2a: 5			OS: 90%
			N2b: 7			
			N2c: 2			
			N3: 0			
						

Weinstein et al. [28] (Nov '10)	47	Oropharynx	T1/2: 36	N/A	26 mths	Overall:
			T3/4: 11			1 yr: 96% (45/47)
						2 yr: 82% (27/33)
			N0: 1			DFS:
			N1: 24			1 yr: 96% (45/47)
			N2a: 1			2 yr: 79% (26/33)
			N2b: 19			
			N2c: 2			
			N3: 0			

White et al. [29] (Dec '10)	89	77 oropharynx (87%)	T1/2: 71	N/A	26 mths (median)	DFS (entire cohort)
		10 larynx (11%)	T3/4: 18			2 years: 86.3%
		2 oral cavity (2%)				
			N0: 27			DFS (primary TORS cohort)
			N1: 8			2 years: 89.3%
			N2a: 11			
			N2b: 26			
			N2c: 12			
			N3: 4			

**Table 3:** Functional and oncologic outcomes for transoral laser microsurgery.

Study	Number of cases	Primary site	Pathological stage	HPV status	Followup period (mean)	Hospital stay (mean)	Tracheostomy dependent (days to decannulation)	Gastrostomy tube dependency	Oncologic outcomes
Steiner et al. [30] (Jan '03)	48	Oropharynx (base of tongue)	T1/2: 13	N/A	47 mths (median)	N/A	0%	6%	5 years:
			T3/4: 35						local control rate: 85%
									recurrence free rate: 73%
			N0: 15						overall survival: 52%
			N1: 9						
			N2: 24						
			N3: 0						

Holsinger et al. [48] (Jul '05)	191	Oropharynx	T1/2: 162	N/A	N/A	9 d	0% permanent	0.5% (1/191)	N/A
			T3/4: 24				3.7% temporary		
			Tx: 5						
									
			N0: 97						
			N1: 50						
			N2: 33						
			N3: 6						
			Nx: 5						

Laccourreye et al. [49] (Jul '05)	166	Oropharynx	T1/2: 147	N/A	10 yrs (median)	N/A	N/A	N/A	Local control rate overall:
			T3: 19						1 year: 91.2%
									5 years: 82.1%
			N0: 85						
			N1: 44						Control rate by T classification:
			N2: 31						T1
			N3: 6						1 year: 98.3%
									5 years: 89.0%
									T2
									1 year: 88.9%
									5 years: 81.7%
									T3
									1 year: 78.9%
									5 years: 62.7%

Rich et al. [52] (Sept '09)	84	Oropharynx	T1/2: 62	HPV-pos: 69	52.6 mths (mean)	4.3 d	11% temporary	1 year: 18.8%	Overall survival
			T3/4: 22	HPV-neg: 4	48.5 mths (median)			5 years: 3.8%	2 years: 94%
				(data for 73 pts)					5 years: 88%
			N0: 3					FOSS-stage 0–2: 81% overall	
			N1: 12						Disease-specific survival
			N2: 64						2 years: 96%
			N3: 5						5 years: 92%

Grant et al. [32] (Dec ‘09)	69	Oropharynx	T1/2: 55	N/A	44 mths (mean)	3 d	0%	0%	Overall survival:
	(treated with surgery only)		T3/4: 14						5 years: 86%
									
			N0: 31						Local control rate:
			N1: 11						5 years: 94%
			N2a: 16						
			N2b: 6						
			N2c: 1						
			N3: 2						

Henstrom et al. [50] (Dec '09)	20	Oropharynx	T1/2: 16	N/A	3.3 yrs (mean)	4.7 d	0% permanent	10% (2/20)	Overall survival:
		(base of tongue)	T3/4: 4		3.2 yrs (median)				2 years: 90.0%
									5 years: 83.1%
			N0: 1						
			N1: 3						Disease specific control rate:
			N2a: 1						2 years: 94.7%
			N2b: 11						
			N2c: 4						

Haughey et al. [31] (Jan '11)	204	Oropharynx	T1/2: 135	HPV-pos: 167	48 mths (mean)	4.4 d	18% temporary	1 year: 18.8%	Overall survival:
			T3/4: 69	HPV-neg: 18	42 mths (median)			5 years: 3.8%	2 years: 89%
				(data available for 185)				(subset data from Wash Uni)	5 years: 78%
			N0: 15						
			N1: 39					FOSS-stage 0–2: 87% overall	Disease-specific survival:
			N2: 135					(203/204)	2 years: 91%
			N3: 15						5 years: 84%

**Table 4:** Gastrostomy tube dependency rates following TORS—long and short term.

Study	Short term	1 Year	2 Years
Weinstein et al. [28] (2010)		2.40%	0%
Moore et al. [14] (2009)	18%	0%	0%
Iseli et al. [33] (2009)		9.50%	
Genden et al. [20] (2011)		0%	0%

**Table 5:** Functional outcomes following TORS for OPSCC.

Study	Number of cases	Primary site	Pathological stage	HPV status	Followup period (mean)	Hospital stay (mean)	Tracheostomy dependent (days to decannulation)	Gastrostomy tube dependency
Weinstein et al. [11]	27	Oropharynx	T1/2: 21	N/A	N/A	N/A	0%	4% (1/27)
(Dec '07)			T3/4: 6					
								
			N0: 4					
			N1: 13					
			N2a:					
			N2b: −10					
			N2c:					
			N3: 0					

Genden et al. [16] (Mar '09)	20 (18 completed cases)	14 oropharynx	T1/2: 20/20	N/A	N/A	1.7 d	0%	0%
		4 supraglottis						
		2 parapharyngeal	N0: 11					
			N1: 6					
			N2: 3					
			N3: 0					

Boudreaux et al. [18] (Apr '09)	36 (29 completed cases)	19 oropharynx (66%)	T1/2: 29	N/A	N/A	2.9 d	0%	Overall: 31%
		7 larynx (24%)	T3/4: 7					oropharynx 3/19 (16%)
		2 oral cavity (7%)						larynx 6/7 (86%)
		1 hypopharynx (3%)	Stage 1: 6 (17%)					oral cavity 0/2 (0%)
			Stage 2: 8 (22%)					hypopharynx 0/1 (0%)
			Stage 3: 3 (8%)					
			Stage 4: 19 (53%)					%)

Iseli et al. [33] (May '09)	54	33 oropharynx	T1/2: 43	N/A	13 mths	N/A	0%	Overall (no previous treatment): 9%
		12 larynx	T3/4: 11					oropharynx 2/33 (6%)
		6 oral cavity						larynx 5/12 (42%)
		3 hypopharynx	N-stage: N/A					oral cavity 1/6 (17%)
								hypopharynx 1/3 (33%)

Moore et al. [14]	45	Oropharynx	T1/2: 33	N/A	12.3 mths	3.8 d	0%	0%
(Nov ‘09)			T3/4: 12				(2–180)	
								
			N0: 7					
			N1: 7					
			N2a: 7					
			N2b: 13					
			N2c: 8					
			N3: 3					

Dean et al. [26] (Apr '10)	36	Oropharynx	T1/2: 36/36	N/A	N/A	1.5 d (TORS primary)	0% primary TORS	0% primary TORS
	15 primary TORS					5 d (TORS salvage group)	0% salvage TORS	0% salvage TORS
	7 salvage TORS					8.2 d (open surgery group)	7% salvage open surgery	43% salvage open surgery
	14 salvage open surgery)							%)

Lawson et al. [19]	24	10 supraglottic	T1/2: 21	N/A	17 mths	9 d	0%	N/A
(Mar ‘11)		10 pharyngeal	T3/4: 1					
		4 oral cavity						
			N0: 13					
			N1: 5					
			N2a: 2					
			N2b: 2					
			N2c: 1					
			N3: 1					

Aubry et al. [15] (Sept '11)	17 (18 tumors)	9 oropharynx	T1/2: 15	N/A	6.5 mths	10 d	0%	12% (2/17)
		7 supraglottis	T3: 3					
		2 hypopharynx						
			N0: 12					
			N1: 4					
			N2a: 0					
			N2b: 2					
			N2c: 0					
			N3: 0					
